# Selective Blocking Effects of 4,9-Anhydrotetrodotoxin, Purified from a Crude Mixture of Tetrodotoxin Analogues, on Na_V_1.6 Channels and Its Chemical Aspects

**DOI:** 10.3390/md13020984

**Published:** 2015-02-12

**Authors:** Noriyoshi Teramoto, Mari Yotsu-Yamashita

**Affiliations:** 1 Department of Pharmacology, Faculty of Medicine, Saga University, Saga 849-8501, Japan; 2 Laboratory of Biomedical Engineering, Graduate School of Biomedical Engineering, Tohoku University, Sendai 980-8575, Japan; 3 Laboratory of Bioorganic Chemistry of Natural Products, Graduate School of Agricultural Science, Tohoku University, Sendai 981-8555, Japan; E-Mail: myama@biochem.tohoku.ac.jp

**Keywords:** 4,9-anhydrotetrodotoxin, LC-FLD techniques, Na_V_1.6 channels, tetrodotoxin

## Abstract

Tetrodotoxin (TTX) is a potent neurotoxin found in a number of marine creatures including the pufferfish, where it is synthesized by bacteria and accumulated through the food chain. It is a potent and selective blocker of some types of voltage-gated Na^+^ channel (Na_V_ channel). 4,9-Anhydrotetrodotoxin (4,9-anhydroTTX) was purified from a crude mixture of TTX analogues (such as TTX, 4-*epi*TTX, 6-*epi*TTX, 11-oxoTTX and 11-deoxyTTX) by the use of liquid chromatography-fluorescence detection (LC-FLD) techniques. Recently, it has been reported that 4,9-anhydroTTX selectively blocks the activity of Na_V_1.6 channels with a blocking efficacy 40–160 times higher than that for other TTX-sensitive Na_V_1.x channel isoforms. However, little attention has been paid to the molecular properties of the α-subunit in Na_V_1.6 channels and the characteristics of binding of 4,9-anhydroTTX. From a functional point of view, it is important to determine the relative expression of Na_V_1.6 channels in a wide variety of tissues. The aim of this review is to discuss briefly current knowledge about the pharmacology of 4,9-anhydroTTX, and provide an analysis of the molecular structure of native Na_V_1.6 channels. In addition, chemical aspects of 4,9-anhydroTTX are briefly covered.

## 1. Introduction

Tetrodotoxin (TTX), one of several marine neurotoxins, was first isolated from pufferfish [1] and subsequently discovered in other marine organisms (such as octopus, flatworms, snails and crabs) as well as small terrestrial animals (newts and frogs). This neurotoxin is a potent and highly selective inhibitor of voltage-gated Na^+^ channels (Na_V_ channels). Since its discovery, TTX has been utilized widely as a pharmacological tool for investigating the biophysical properties of Na_V_ channels [2]. TTX selectively blocks the pore of the Na_V_ channel from the extracellular side of the plasma membrane, thereby preventing the influx of Na^+^, without affecting other ion channels and receptors [3].

4,9-AnhydroTTX, which possesses a weaker toxic action than other TTX analogues, was first isolated from pufferfish liver using high performance liquid chromatography (HPLC; [4]). In comparison with the potency of TTX, 4,9-anhydroTTX was found to exhibit weaker blocking effects on Na_V_ channels in squid axon [5] and lower affinity binding to rat synaptic membrane preparations containing Na_V_ channels [6]. Subsequently, 4,9-anhydroTTX has been quantified using liquid chromatography fluorescence detection (LC-FLD; [7]) and liquid chromatography mass spectrometry (LC/MS) methods [8,9,10,11,12]. However, details of the inhibitory effects of 4,9-anhydroTTX on the activity of Na_V_ channels have remained elusive. Using electrophysiological techniques, heterologous expression studies of seven different types of Na_V_1.x channels (Na_V_1.2–Na_V_1.8 channels) in *Xenopus* oocytes revealed that 4,9-anhydroTTX was a highly selective blocker of the Na_V_1.6 channel isoform, with a blocking efficacy 40–160 times higher than for other TTX-sensitive Na_V_1.x channel isoforms, suggesting that 4,9-anhydroTTX could be an invaluable tool for identifying Na_V_1.6 channel-mediated functions [13]. It has been reported recently that Na_V_1.6 channel-mediated resurgent-like Na_V_ currents recorded in freshly isolated smooth muscle cells of the mouse vas deferens, were reversibly abolished by 4,9-anhydroTTX [14]. This observation strongly suggests that 4,9-anhydroTTX inhibits the channel activity of native Na_V_1.6 channels with a similar potency to that seen in Na_V_1.6 channel expression studies [13]. Classically, Na_V_ channels have been classified into TTX-sensitive and TTX-resistant types, according to the potency and selectivity of TTX [2]. We believe that the development of selective TTX analogues that act on specific Na_V_ channels, and hence show minimal toxicity, will provide highly useful pharmacological tool and be of great therapeutic interest.

In this review, we will focus on the target Na_V_ channels for 4,9-anhydroTTX (*i.e.*, Na_V_1.6 channels), and discuss the selective blocking effects of 4,9-anhydroTTX on Na_V_1.6 channel-mediated signals. In addition, certain chemical aspects of 4,9-anhydroTTX will be briefly covered.

## 2. Na_V_ Channels

Na_V_ channels are members of an ion channel protein superfamily that are widely expressed in both neuronal and non-neuronal cells. The essential role of Na_V_ channels is in the generation of the action potential upstroke (*i.e.*, the initiation and propagation of action potentials) in excitable cells (such as nerve fibers, skeletal muscle fibers, cardiac myocytes and some smooth muscle cell types). Na_V_ channels also influence subthreshold electrical activity through persistent and resurgent Na_V_ currents [15]. Additionally, recent studies have revealed that certain Na_V_ channels may be involved in the processes underlying normal and pathological pain [16], and that they may be up-regulated in cancer, in general favoring invasive/metastatic phenotypes [17]. Thus, it is generally believed that Na_V_ channels play important roles in various physiological and pathophysiological conditions [18].

**Figure 1:**
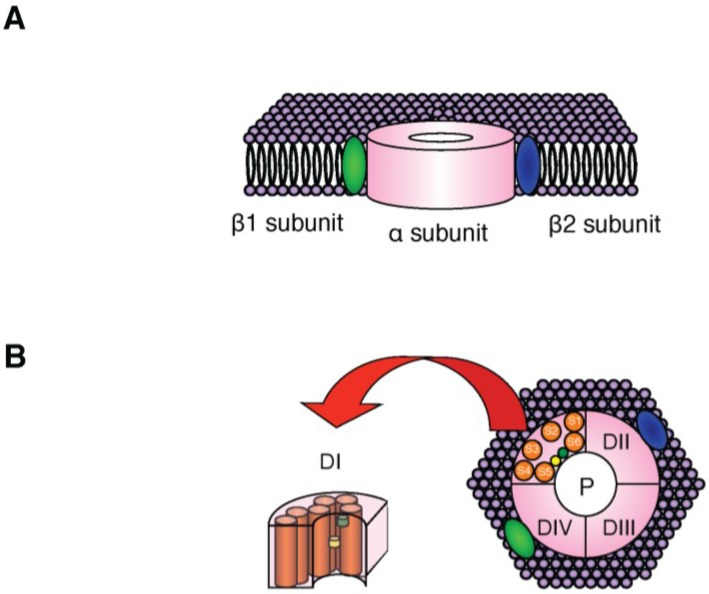
Molecular structure of Na_V_ channels. (**A**) Schematic illustration of the predicted topology of Na_V_ channels (α subunit and β subunits). The transmembrane domain model proposed by Catterall (2000) is shown [18]; (**B**) The α subunit is formed by four homologous domains (DI–DIV). Each domain consists of six α-helical transmembrane segments (S1–S6). P: channel pore.

In the 1980s, two proteins with substantially different biochemical properties (*i.e.*, 260 kDa and 30–40 kDa) were detected in Na_V_ channels isolated from mammalian skeletal muscle and brain [19,20]. Later cloning and heterologous expression studies revealed that the α subunit (260 kDa) forms the core of the channel (*i.e.*, the channel pore) and is responsible for voltage-dependent gating, ionic selectivity and TTX-binding, and that the β subunits (30–40 kDa) act as auxiliary subunits to modify channel function [21]. As shown in Figure 1, the molecular structure of Na_V_ channels comprises one α subunit and one or more β subunits.

To date, nine genes (*Scn1a*–*5a* and *Scn8a*–*11a*, comprising highly homologous clones) encoding the α subunits of TTX-sensitive (Na_V_1.1–Na_V_1.4, Na_V_1.6 and Na_V_1.7 channels, respectively) and TTX-resistant (Na_V_1.5, Na_V_1.8 and Na_V_1.9 channels, respectively) Na_V_ channels have been identified within a single family of Na_V_ channels, Na_V_1.x channels (Table 1; [22]). Two other genes (*Scn6a* and *Scn7a*) encoding Na_X_ (also known as NaG or Na_V_2.1 channel) have also been identified; these have functional motifs that are related to Na_V_ channels (such as the voltage-sensing and interdomain regions), but not the Na_V_ channel pore itself (these channels have not been functionally expressed as yet) [22]. The latter isoforms are thought to be involved in the transport or absorption of Na^+^, by sensing the Na^+^ concentration gradient across the plasma membrane; therefore, they have been suggested to act as a “concentration-sensitive” Na^+^ influx protein [23,24].

**Table 1:** Location and distribution of Na_V_ channel genes. S, sensitive; R, resistant; CNS, central nervous system; PNS, peripheral nervous system; SMCs, smooth muscle cells.

α Subunit (Channel pore)	Coding gene	TTX-Sensitivity	Predominant location
NaV1.1	*Scn1A*	S	CNS, PNS
NaV1.2	*Scn2A*	S	CNS
NaV1.3	*Scn3A*	S	CNS (embryonic)
NaV1.4	*Scn4A*	S	Skeletal muscle
NaV1.5	*Scn5A*	R	Heart muscle
NaV1.6	*Scn8A*	S	CNS, PNS, Glia, Nodes of Ranvier, SMCs
NaV1.7	*Scn9A*	S	PNS, Schwann cells
NaV1.8	*Scn10A*	R	PNS (sensory neurons)
NaV1.9	*Scn11A*	R	PNS

**Figure 2:**
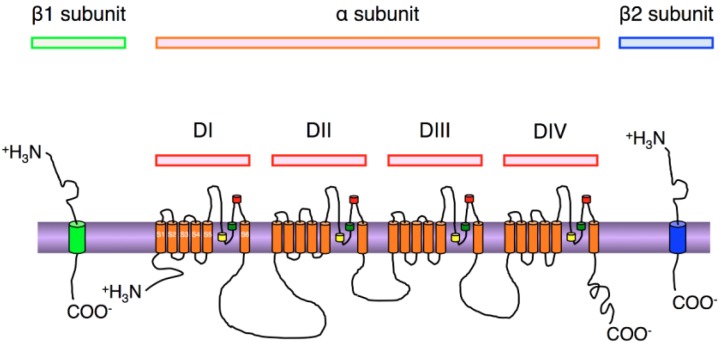
Schematic representations of the α and β subunit s of Na_V_ channels. The α subunit is formed by four homologous domains (DI–IV). Each domain consists of six α-helical transmembrane segments (S1–S6). P loops are located between segment 5 and 6, which are the pore-lining regions (yellow and green). TTX binding sites are shown in red. The β subunits consist of an N-terminal containing an immunoglobulin-like loop and the splice site, a *C*-terminal, and a membrane-spanning segment.

Functional expression of the α subunit is required for the formation of the channel pore and ion selectivity filter, and this subunit determines several biophysical properties of the Na_V_ channel [18]. As shown in Figure 2, the α subunit has four homologous domains (DI–DIV), each containing six transmembrane α-helical segments (S1–S6), which are connected by extracellular and intracellular loop regions. Specific amino acid sequences of the α subunit form the channel pore, voltage sensor, inactivation gates and various phosphorylation sites [25]. It is generally believed that TTX binds within the outer vestibule of the Na_V_ channel to multiple residues in the α subunit that controls Na^+^ permeation (the P loop; between S5 and S6 in each domain is a short segment that appears to fold half-way into the membrane, like a hairpin structure); furthermore, TTX is thought to block the influx of Na^+^ by occluding the outer pore [26].

The β1–β4 isoforms of the β subunits are encoded by four genes (*Scn1b*–*4b*), with two splice variants of *Scn1b* (*Scn1b*-a and *Scn1b*-b) encoding β1-A and β1-B, respectively. The β subunits share a common structure [27] consisting of a single membrane-spanning domain, a small intracellular *C*-terminal domain, and a large extracellular *N*-terminal domain incorporating an immunoglobulin-like fold that is similar to that found in cell adhesion molecules (Figure 1; [21]). Recent studies have revealed that the β subunits play significant roles in the regulation of channel localization and in interactions with cell adhesion molecules, the extracellular matrix, intracellular cytoskeletal proteins and other molecules that modulate cell migration and aggregation [28].

## 3. Localization of Na_V_1.6 Channels

Approximately two decades ago, analysis of a transgene insertion site led to the isolation of a novel Na_V_ channel gene designated *Scn8a* [29]. *Scn8a* encodes a 1732-amino acid member of the α subunit gene family of Na_V_ channels (*i.e.*, the Na_V_1.6 channel). Expression of *Scn8a* has been shown in the brain and spinal cord [29], with a widespread distribution in many regions of the central nervous system (CNS), including cerebellar granule cells, and pyramidal and granule cells of the hippocampus [30]. The human *Scn8a* gene has been mapped to a conserved linkage group on chromosome 12q13, and is a candidate gene for inherited neuromuscular disease [29].

The Na_V_1.6 isoform was initially detected in the nodes of Ranvier in the peripheral nervous system (PNS) and CNS [31,32], and along non-myelinated axons [33], suggesting that this type of Na_V_ channel plays an important role in the function and regulation of both the PNS and CNS. Na_V_1.6 channels have been proposed to mediate resurgent and persistent Na^+^ currents with a resulting effect on repetitive firing behavior [34]. The Na_V_1.6 channel is also widely distributed throughout the spinal cord [35]. Its expression in the dorsal root ganglion (DRG) is predominantly in large myelinated A-fiber neurons [36]. It has been reported that Na_V_1.6 channels are also expressed in the axons that comprise small nerve bundles underlying the epidermis, and in epidermal free nerve terminals, including nociceptors [37]. Furthermore, Na_V_1.6 channels are expressed in keratinocytes, which may contribute to the sensation of pain. It is noteworthy that a significantly increased expression of Na_V_1.6 channels has been found in human skin biopsies taken from patients with complex regional pain syndrome and post-herpetic neuralgia [38]. These findings suggest a major role for Na_V_1.6 channels in the function of small-diameter sensory nerve endings and the related pathophysiology. In addition, several studies have provided strong evidence that the Na_V_1.6 channel is the predominant Na_V_ channel isoform expressed in microglia, which are known to play an important role in pathological pain, and that Na_V_1.6 channels contribute to the response of microglia by multiplying various activating signals [39].

## 4. Na_V_1.6 Channel-Null Mice (*med* Mice) Lacking Expression of *Scn8a*

The loss of *Scn8a* expression in the mouse represents a unique and useful animal model for studying Na_V_1.6 channel-mediated phenomena (including ethology, functional analysis and morphological studies). It has been reported that mutations at the “motor endplate disease” (*med*) locus on distal chromosome 15 of the mouse results in a recessive neuromuscular disorder [40]. Analysis of a transgene-induced mutation at the mouse *med* locus was performed to identify the Na_V_1.6 channel gene, *Scn8a* [29]. The original *med* mutation (C3HeB/FeJ-*Scn8a^med^*) arose in Edinburgh, Scotland in 1958, and the *med^J^* allele (C3HeB/FeJ-*Scn8a^med^*^/*J*^) was identified at The Jackson Laboratory in the USA [41]. The *med* mutation is caused by the insertion of a truncated long interspersed element (LINE) into exon 2 of *Scn8a*. The *med* transcript is spliced from exon 1 to a cryptic acceptor site in intron 2, whilst a 4 base-pair deletion within the 5′ donor site of exon 3 in the *med^J^* allele results in splicing from exon 1 to exon 4. Both mutant transcripts possess altered reading frames with premature stop codons, which are close to the protein N terminus. The loss of *Scn8a* expression leads to progressive paralysis and early death.

Homozygous mice with a Na_V_1.6 null allele (*med* mice, Na_V_1.6^−/−^) have been widely utilized to investigate the molecular and functional contributions of Na_V_1.6 channel-mediated mechanisms. The *med* and *med^J^* mutations both result in severe, progressive skeletal muscle atrophy, secondary to a loss of the functional innervation [42,43], that is lethal within three to four weeks of birth [41]. Although the number of motor neurons in the spinal cord of mutant mice is not reduced, transmission of excitatory activity across the neuromuscular junction is impaired. The *med* allele also produces a cerebellar ataxia, which is associated with a loss of spontaneous electrical activity in cerebellar Purkinje cells [44]. In smooth muscle, the electrophysiological properties of Na_V_ currents through Na_V_ channels have been compared between Na_V_1.6-null mice (Na_V_1.6^−/−^) lacking the expression of *Scn8a* and their wild-type littermates (Na_V_1.6^+/+^), leading to the conclusion that the smooth muscle-type Na_V_ channel is likely to be Na_V_1.6 [14,45,46].

Pharmacological inhibition of Na_V_1.6 channel activity represents an alternative to the Na_V_1.6 channel-null mouse model. 4,9-AnhydroTTX is a highly selective toxin for the Na_V_1.6 channel, with an *IC_50_* for this isoform that is 40–160 times lower than that for other TTX-sensitive Na_V_1.x isoforms (*IC_50_* = 7.8 nM for the Na_V_1.6 channel, compared with 1260 nM for the Na_V_1.2 channel, 341 nM for the Na_V_1.3 channel, 988 nM for the Na_V_1.4 channel and 1270 nM for the Na_V_1.7 channel) [13]. Although much higher concentrations of 4,9-anhydroTTX (>300 nM) will inhibit the activity of other Na_V_1.x channels, appropriate concentrations of 4,9-anhydroTTX are considered to represent a convenient and selective pharmacological tool for assessing the function of the Na_V_1.6 channel.

Much higher concentrations of 4,9-anhydroTTX are required to inhibit the activities of TTX-insensitive Na_V_ channels (*i.e.*, Na_V_1.5 and Na_V_1.8 channels) compared with those needed to inhibit TTX-sensitive Na_V_ channels [13]. Based on the available observations, it is reasonable to propose that 4,9-anhydroTTX likely blocks Na_V_ channels through the same site(s) and inhibitory mechanism(s) as TTX. However, the exact binding site(s) for 4,9-anhydroTTX in Na_V_1.6 channels remain(s) elusive [13]. Further studies, examining the amino sequence alignments of the binding sites, are needed to characterize the mechanisms underlying the sensitivity to 4,9-anhydroTTX.

## 5. Chemical and Toxicological Aspects of 4,9-AnhydroTTX

**Figure 3:**
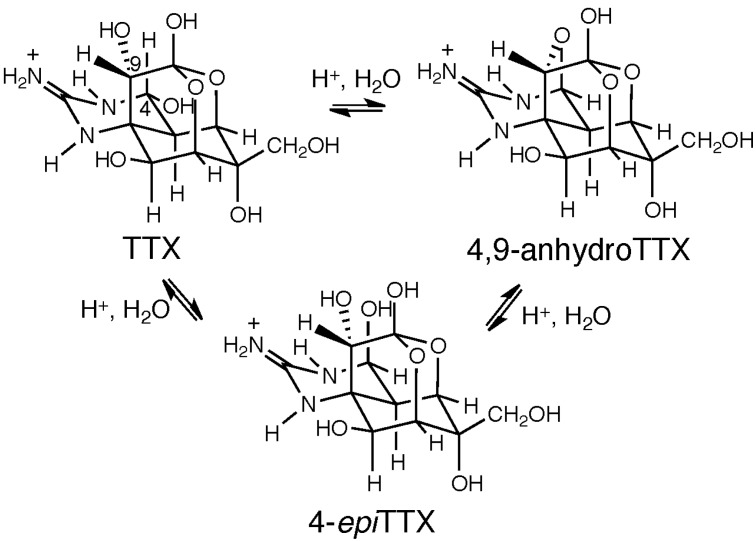
The interconversion of TTX, 4-*epi*TTX and 4,9-anhydroTTX resulting from the chemical equilibrium.

TTX [47,48,49] is usually present as a mixture of TTX, 4-*epi*TTX and 4,9-anhydroTTX in an acidic aqueous solution, due to the existence of a chemical equilibrium between these compounds (Figure 3). It has been reported that both TTX and 4,9-anhydroTTX epimerize slowly to give an equilibrium mixture of TTX and 4,9-anhydroTTX in a ~4:1 ratio (NMR) [49]. As a result, the maximal purity of 4,9-anhydroTTX assessed by LC-FLD is ~99% [14], since small amounts of both TTX (0.5%) and 4-*epi*TTX (0.5%) are present due to their conversion from 4,9-anhydroTTX under acidic conditions. NMR data for TTX [50,51] and 4,9-anhydroTTX [4,52] have been reported. The major difference between TTX and 4,9-anhydroTTX is in the ^3^*J*_H4/H4a_ value, which is 10 Hz for TTX and 0 Hz for 4,9-anhydroTTX. An LC-FLD approach for the quantitation of TTX and 4,9-anhydroTTX has been described [7], whilst we have developed an alternative LC/MS method [8,9,10,11,12].

Both 4-*epi*TTX and 4,9-anhydroTTX were successfully isolated from the pufferfish [4], and their toxicities determined in mice (ddY strain, male, 19–20 g, intraperitoneal administration; TTX: 4500 mouse units (MU)/mg; 4-*epi*TTX: 710 MU/mg; 4,9-anhydroTTX: 92 MU/mg). We have also determined the affinities of these compounds in rat brain membranes, based on a competitive binding assay using ^3^H-saxitoxin; the dissociation constants of 4-*epi*TTX and 4,9-anhydroTTX were 38- and 100-fold larger, respectively, than that of TTX [6].

It has also been reported that conversion between TTX, 4-*epi*TTX and 4,9-anhydroTTX occurred in living, cultured, juvenile pufferfish (kusafugu, *Takifugu niphobles*) [53]. The latter authors separately administered almost pure TTX, 4-*epi*TTX or 4,9-anhydroTTX to the test fish by intramuscular injection. When pure TTX was administered, the ratio of TTX to 4,9-anhydroTTX was ~5:4 (mol/mol) 4 days after injection. Moreover, administration of pure 4,9-anhydroTTX resulted in a ~1:5 ratio (mol/mol) of TTX to 4,9-anhydroTTX, suggesting that TTX is rapidly converted to 4,9-anhydroTTX in living pufferfish, whereas conversion of 4,9-anhydroTTX to TTX occurs at a somewhat slower rate.

**Figure 4:**
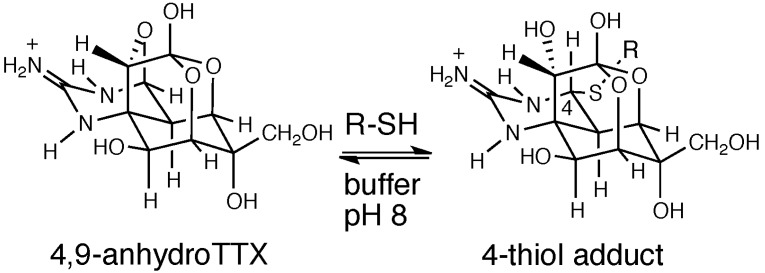
Formation and hydrolysis of the 4-thiol adduct of TTX.

It has been reported previously that 4,9-anhydroTTX can react with a large excess of thiol compounds, such as cysteine, reduced glutathione (GSH) and mercaptoethanol (Figure 4; [54]). The hemiaminal ether carbon C-4 present in 4,9-anhydroTTX can easily accept nucleophiles. In fact, the cysteine adduct of TTX, 4-*S*-cysteinylTTX, has been isolated from the liver of the pufferfish, *Takifugu pardalis*, as a probable metabolite of TTX. This finding suggests that there is an abundance of 4,9-anhydroTTX and cysteine in the pufferfish liver. Indeed, the ratio of 4,9-anhydroTTX to total TTX analogues in the liver of the pufferfish is significantly larger than that in the ovary, although the reason for this specific distribution of 4,9-anhydroTTX in the liver remains unknown. The nucleophilic addition of thiols to the C-4 in 4,9-anhhydroTTX proceeds easily under slightly alkaline conditions (pH 8.0) depending on the pKa of the thiol groups (pKa of cysteine, 8.3–8.5). However, TTX barely reacts with thiol compounds, probably because the hemiaminal carbon (C-4) in TTX is less reactive than that in 4,9-anhydroTTX; a 4,9-ether would likely be a better leaving group than a 4-hydroxy group. In addition, the thiol adducts of TTX, 4-*S*-cysteinylTTX and 4-*S*-glutathionylTTX (4-GSTTX), are not stable under near-physiological conditions, with hydrolysis to 4,9-anhydroTTX in the presence of thiol compounds occurring readily in 0.8 M sodium phosphate buffer at pH 8 and 25 °C. Therefore, detoxification of TTX with thiols may not prove to be successful, even though 4-*S*-cysteinylTTX is almost non-toxic to mice.

It is notable that the total chemical synthesis of TTX analogues [55] usually involves the generation of 4,9-anhydro compounds until nearly the final step; the 4,9-anhydro compounds are then derived to the relevant TTX analogues by hydrolysis of the 4,9-ether bonds in aqueous trifluoroacetic acid. This reaction arises because the 4,9-ether bonds stabilize TTX-related molecules, allowing them to survive different reaction conditions as these complex molecules are synthesized. Similarly, we have predicted recently that 4,9-anhydroTTX is the immediate precursor of TTX in the biosynthesis of TTX, although the total TTX biosynthetic pathway has not yet been fully elucidated [52].

## 6. Concluding Remarks

The present review has provided information concerning 4,9-anhydroTTX, a TTX analogue purified from crude TTX, and described the molecular and physiological properties of its target Na_V_1.6 channel. It is well known that Na_V_1.6 channels play an important role in pain and the development of cancer and Na_V_1.6 channels have also been implicated in both normal and pathological pain [17]. In future, highly selective agents for Na_V_1.6 channels may prove clinically useful in the relief of pain and the prevention of cancer growth. Based on the findings to date, it is plausible that 4,9-anhydroTTX, a highly isoform-selective TTX analogue, will turn out to be an invaluable research tool not only for the identification of Na_V_1.6 channel-mediated functions but also the development of drugs for therapeutic interventions. However, 4,9-anhydroTTX may contain a small amount of TTX due to chemical equilibrium when kept in solution. TTX analogues show promise as tools for clinical research, but conclusive results have yet to be published. Further studies are required to determine the stability of 4,9-anhydroTTX in a crude mixture of TTX analogues.
